# “To obey or not to obey” - Medical students’ response towards professional dilemmas in a hierarchical and collectivist culture

**DOI:** 10.1371/journal.pone.0261828

**Published:** 2021-12-23

**Authors:** Diantha Soemantri, Nadia Greviana, Ardi Findyartini, Tiara Berliana Azzahra, Kemal Akbar Suryoadji, Rita Mustika, Estivana Felaza

**Affiliations:** 1 Faculty of Medicine, Department of Medical Education, Universitas Indonesia, Jakarta, Indonesia; 2 Faculty of Medicine, Medical Education Center, Indonesian Medical Education and Research Institute (IMERI), Universitas Indonesia, Jakarta, Indonesia; 3 Faculty of Medicine, Undergraduate Medical Program, Universitas Indonesia, Jakarta, Indonesia; International Medical University, MALAYSIA

## Abstract

**Background:**

Clinical clerkship programme in medical schools were developed to provide students with direct interactions with patients and observe clinical teachers in practice. However, professional dilemmas are prone to occur due to the nature of experiential learning. Several studies across different cultures showed that medical students responded differently towards professional dilemma.

**Aims:**

This study aims to explore how medical students respond to professional dilemmas occurred during their clinical clerkships and to what extent culture influences the responses.

**Method:**

A qualitative descriptive approach was used in this study. We conducted four focus group discussions with final year medical students who were selected using maximum variety sampling method. Thematic analysis was conducted following the transcription of the focus groups.

**Results:**

We identified the impact of dilemmas on students’ emotions and concerns, students’ responses towards professional dilemmas, and factors affecting responses to dilemmas in clinical clerkship, which confirmed that cultures played roles in how students responded towards professional dilemmas.

**Conclusion:**

This study has identified that culture, to some extent, influenced the way students responded to professional dilemmas. Therefore, it is paramount to develop a conducive and culturally sensitive educational environment and students’ ability to learn from professional dilemma experienced in the workplace for developing their professional identity.

## Introduction

Medical students require adequate exposure to workplace-based patient management provided in clinical clerkship [1]. The clinical clerkship programme has adapted social cognitive theory, in which students learn through exposure to authentic and challenging experiences, such as those involving direct patient care, interacting with other health professionals, and sharing their values. Students also learn about professionalism through direct interactions with patients, while periodically observing physicians in practice as role models [2,3]. Such experiential learning involves not only cognitive aspect, but also feeling and emotions [4]. Therefore, professional dilemmas are prone to occur.

A professional dilemma is a situation in which students observed or were involved in behaviours that were against their values of which they perceived as unprofessional [5], including experiences related to patient encounters, mistreatment, and medical culture [6]. Monrouxe and Rees (2012) identified five categories of professional dilemmas based on 200 medical students’ narratives of professionalism during clinical clerkships: (1) consent dilemmas, (2) patient safety and dignity breaches by medical students, (3) patient safety and dignity breaches by healthcare professionals, (4) students’ identity dilemmas, and (5) abuse dilemmas [7].

Despite professional dilemmas that occurred in the workplace, development of professionalism, showed by sets of attributes such as competence, altruism, morality, and integrity, has always been the foundation of medical education [8]. As professionalism are acquired mainly through the process of interactions and socializations within the community of practice, supportive and nurturing learning environment is essential in promoting student professionalism [9,10].

Professional dilemmas experienced by medical students in the workplace may cause diverse reactions. In situations where medical students identified an appropriate response to a particular situation but were restrained from enacting the response, moral distress occurred [11,12]. A multicentre study using the behavioural explanations approach [13] conducted by Rees and Monrouxe (2011) discovered that medical students showed two types of responses to professional dilemmas of learning to conduct intimate examinations without valid consent [14]. The two responses were compliance and resistance, each with several behavioural explanations. The behavioural explanations approach was used to explore how people try to understand behaviours and events happening around them and how they legitimately respond to them. The study identified three explanation modes for intentional behaviour: reason explanation, referring to individual mental state (belief, value, or desire); causal history of reason, referring to particular background distance from oneself during deliberation of behaviour; and enabling factor, which refers to external factors [14].

Ginsburg et al. (2003) identified two main approaches taken by undergraduate medical students of a Canadian university when faced with a professional dilemma, based on the principle of avowed (where students maintain their professionalism values) and unavowed principles [15]. Ho et al. (2012), who conducted a similar study in a Taiwanese setting, found generally comparable results with several notable differences in the influencing factors, such as the importance of following the advice of seniors and consideration of the impact of their behaviour on multiple social relationships [16]. In an Italian setting, Consorti et al. (2019) demonstrated that following seniors’ advice and reporting inappropriate behaviour were not identified as possible responses from the students [17]. It is argued that students’ responses to professional dilemmas are somewhat influenced by cultural norms.

As values and other cultural aspects played significant roles in professional dilemmas and how students responded to them, it was important to explore these in various settings [7,17]. According to Hofstede’s cultural dimensions [18], students in higher power distance societies tend to feel discouraged from speaking up [19]. Collectivist culture prioritises connectedness, and personal actions are usually affected by others in society [20]. Security in career and stability are known to be important issues for societies with high uncertainty avoidance [21]. Apart from those cultural dimensions, some cultures were also known to be more restrained in that they put less emphasis on their own desire for happiness compared to Western countries [22]. Therefore, this study is aimed at exploring how medical students in high power distance and collectivist culture responded to professional dilemmas that occur during their clinical clerkships, and to what extent culture influences the responses. The findings of this study are expected to provide insights for medical educators, especially those with similar cultural determinations, to be aware of the risk of professional dilemmas encountered by medical students in the workplace and to provide further support in promoting professional development among medical students and enable learners to learn from the dilemmas.

## Methods

### Context

The present study was conducted at a public medical school in Indonesia. Indonesia is a country with high power distance, high collectivism and high uncertainty avoidance [18]. Although Indonesia is a Muslim majority country, as one of the eldest medical schools in Indonesia, the school receive students with various religion, ethnicity, and socio-economic status. The undergraduate programme at the school accepts high-school leavers (17–19 years old) and implements a competency-based curriculum and is divided into preclinical and clinical clerkship stages. Bahasa Indonesia is the main language used to communicate among students, with teachers, and patients in both stages. Clinical clerkship is conducted in year four to year six of their study, in state teaching hospitals, both national and district hospitals, and also primary care facilities to provide students with sufficient exposure to various cases relevant to their competencies. Students graduate as medical doctors after completing all clinical rotations and passing the national board examination in the sixth year of medical school, mostly in their early twenties.

### Design and data collection

A qualitative descriptive approach [23] was used in this study to facilitate in-depth exploration of how medical students responded to professional dilemmas encountered during their clinical clerkships. Data collection for qualitative study used in this study was focus group discussions. Prior to the discussions, focus group schedule was created with lists of probing questions that allowed students to reflect on dilemmatic experiences occurred to them, either through direct involvement or observation and recount how they responded to each dilemma and what made them enact such responses. Probing questions on professional dilemma was developed according to Monrouxe & Rees (2012) [7] framework as the opening questions and further questions on how students responded to mentioned dilemma were developed according to the aforementioned studies [11–14]. Moreover, all focus groups moderators gathered to ensure similar understanding about all probing questions before focus groups took place.

Thirty-seven of the sixth-year students of clinical clerkship who were enrolled in their last rotation module of the clinical clerkship consented to participate in focus groups. A maximum variety sampling strategy was applied to ensure representativeness of study respondents [24], taking into account characteristics of respondents: students’ gender and academic achievement (GPA) in each focus group, so that it captured different perspectives regarding responses to professional dilemmas. We attempted to mix the characteristics of respondents in each group, with 5–6 male participants and 4 female participants in each group as well as variation of GPA among participants.

Focus group discussions were done to less than ten respondents in each group to ensure the optimum data gathering. Each focus group was moderated by medical educators who were familiar with qualitative study and were not directly involved in the teaching, learning, or assessing students within the clinical clerkship. We first started with two focus groups and data saturation was reached in the fourth focus group. All focus group discussions were conducted in face-to-face interaction in November 2019.

Participations of this study was voluntary. Students who participated in this study were informed that their identity would be kept confidential and that any data obtained from this study would not affect their future performance or grades. Students signed written consent forms prior to the focus groups.

### Data analysis

All four focus groups were audio recorded. As in qualitative study, data analysis was conducted in parallel with the data collection since the main researchers acted as focus group moderators [25]. All focus groups recordings were transcribed verbatim. The data obtained was analysed thematically using the steps for coding and theorisation (SCAT) method [26]. All data regarding responses to professional dilemmas were then re-grouped into categories according to the behavioural explanations theory approach as applied in the previous study by Rees and Monrouxe (2011) [14].

This study was approved by the Institutional Review Board (number KET1139/UN2.F1/ETIK/XI/2017).

## Results

Based on the four focus groups involving 21 and 16 male and female students, respectively, with varied academic achievement (GPA between 3.34 and 3.83, out of 4). We attempted to mix the characteristics of respondents in each group, with 5–6 male participants and 4 female participants in each group as well as variation of GPA among participants. We identified the impact of dilemmas on students’ emotions and concerns, students’ responses towards professional dilemmas and factors affecting responses to dilemmas in clinical clerkship. The identified themes are shown in Table 1.

**Table 1:** Impact, response, and factors related to professional dilemmas in medical students.

No	Identified themes and subthemes	Numbers of quotes
1	Impact of dilemmas on students’ emotions and concerns	72
2	Responses towards professional dilemmas	
	1. Compliance	54
	2. Resistance	44
3	Factors affecting responses to dilemmas:	
	1. Reaction of authority figures towards dilemmas	29
	2. Existence of clear reporting and follow-up mechanism	27
	3. Future implications of students’ responses	21
	4. Perceptions of dilemma and professionalism	33
	5. Hierarchical relationships in the workplace	22

While examining the types of dilemmas were not included as the study objective, to set the context of the results and discussion, some examples of dilemmas as perceived by the students and implied within the discussion were those regarding students’ identity, doctor-patient communications, preceptor- student or junior-senior relationships, and irrelevant tasks for students.

### Impact of dilemmas on students’ emotions and concerns

According to Table 1, the professional dilemmas that occurred during the clinical clerkship raised the respondents’ concerns and impacted them emotionally. Feelings of anxiety, confusion, fear, apathy and demotivation are mentioned by respondents in response to professional dilemmas during their clinical years.

*‘[Experiencing] it really affected my confidence*.*’ [FGD 1-E*]

*‘It was really stressful witnessing [the professional dilemma]*, *especially when it involved the preceptor and patient*.*’ [FGD 3- L*]

These concerns and feelings resulted in silent behaviour and failure to report the dilemmas. Given the distressed feelings following dilemmatic experiences, some respondents decided to avoid such experiences in the future. Other respondents mentioned how talking about the experience and sharing with peers was helpful for them in coping with dilemmas.

*‘I would just avoid it*, *make sure we don’t have to meet [the same situation again]*.*’ [FGD 3-U*]

*‘And to cope with the stress*, *I just talked to my friends and we can finally laugh about it or looking for other entertainment*.*’ [FGD 2-*Q]

### Responses towards dilemma

#### Compliance

The most common response to professional dilemmas used by the respondents was to comply with the situation or instructions. Respondents complied with the dilemmatic instructions from their preceptors, residents, or other more senior health professionals they work with during their clinical clerkship. Some respondents described how they decided to comply as they tried to “put themselves in other people shoes” towards people involved in the situations.

*‘When I see seniors [enacted certain behaviours]*, *I tried to dive into the situation [trying to understand] as that was how they cope [with the situation]*.*’ [FGD 1-G*]

Some other respondents expressed “normalising the situations” behaviour, as they said it was not uncommonly seen in their workplace.

*‘In my first rotation*, *I was exposed [to the professional dilemma]*, *and I comply to that behaviour because it seemed as a common habit there*.*’ [FGD 2-M*]

Another form of compliance shown in this study was that respondents also tried several strategies to fulfil their learning goals, despite the dilemma.

*‘Sometimes when (s)he [a superior] asked*, *‘True*, *right*?*’ We just answer ‘yes’ [to be able to participate in the learning activity]’ [FGD 3-W*]

*‘Being exposed to that situation*, *I have to be honest that I often obey [with the instruction]*. *Although I was not sure whether it’s a right thing to do*.*’ [FGD 1-D*]

#### Resistance

Resistance against the dilemma was the least frequently mentioned response. Only few respondents mentioned their experience of directly refusing and confronting while being asked to perform or experiencing dilemmatic behaviour. Those who mentioned resistance behaviour mentioned how they tried to maintain their professional values.

*‘I witnessed a friend who refused because she doesn’t want to [as it was against her value and idealism]*. *But thank God the senior understood and said ‘I’ll just do it’*. *So (s)he did it by him(her)self*.*’ [FGD 3-U*]

Some respondents tried to fix the situation through direct discussions with their supervisor or reporting the situation to the programme authorities.

*‘For me*, *I tried to approach the person [who ordered me to do something] and discussed what my point of view and knowledge regarding the matter [although it was different to what was ordered]*.*’ [FGD 3-Z*]

*‘We tried reporting the problem to the unit [that is in charge]*.*’ [FGD 1-G*]

Learning through negative role modelling was also mentioned by students in resisting professional dilemmas.

*‘We cannot change how ones behave*, *but the things we can control [are] how we behave*, *how we change our attitude towards others*, *and make sure that we don’t do the same things like them*.*’ [FGD 2-O*]

### Factors influencing response to professional dilemma

The current study also showed several factors that influenced the participants’ choices of response to professional dilemmas, including the reaction of authority figures, the existence of reporting and follow-up mechanisms of professional dilemmas, future implications of students’ responses, perceptions of dilemma and professionalism and hierarchical relationships in the workplace.

#### Reaction of the authority figures

Respondents stated that the reaction of some teachers, especially those who were in charge as administrators, played roles in determining how they responded to professional dilemmas.

*‘I really appreciated it when we reported the problem and receive a responsive cue from the administrator and some measures were taken to fix the situation*.*’* [FGD 2-O]

#### Existence of clear reporting and follow-up mechanism

The existence of clear reporting and follow-up mechanisms was mentioned as critical for respondents’ coping mechanisms in professional dilemmas. Some respondents would have reported the situation if they had known who they could report to. Impactful follow-up mechanism following previous reports also strengthen their decision to speak up in the future.

*‘I had once reported the problem to the [administrators]*, *but the response was “Oh*, *that happens all the time*.*”‘* [FGD 4-CY]

#### Future implications of students’ responses

In responding to professional dilemmas, respondents seriously considered the implications of their responses for their study in the future. Respondents were not only concerned about how their actions might affect their grades and future endeavours, but also how others (other students, juniors, seniors and health professionals) and their relationships with them might be affected by their responses.

*‘We were afraid if we reported*, *later on*, *something will happen to the senior or we will not have good relation with the senior*, *or later on we will become a senior too or eventually we will be colleagues and it will make things become uncomfortable*.*’* [FGD 4 –CZ]

#### Perceptions of dilemma and professionalism

Respondents’ perceptions of the dilemma and professionalism influenced how the respondents viewed the event itself and whether it was harmful or not. This perception was also influenced by their cultural background, upbringing and socialisation.

*‘In my opinion*, *as long as it doesn’t cause major impact to the victim or student*, *it’s okay to comply [to the dilemma]*.*’* [FGD 2–S]

*‘It really depends on someone’s ethnical background and the place where (s)he grows up*, *how his/her friends usually behave*, *the customs really determine how (s)he behaves*.*’* [FGD 3-V]

Response towards dilemmas were also affected by the context of the situation itself. In some situations, some behaviour, such as senior healthcare professionals instructing students to complete tasks unrelated to or beyond their learning scope, was perceived to be common, regardless of the professionalism concept.

*‘I realise that it is not just one person who did that*, *but all of them are the same*.*’* [FGD 4-CX]

*‘We just talked about the unpleasant experience we had*, *…*. *we want to do something about it*, *but it is difficult to make a change because it is already become a custom*, *inherited from one person to another*. *So I start from myself by not doing such thing*.*’* [FGD 2-L]

#### Hierarchical relationships in the workplace

Another factor affecting respondents’ coping mechanisms was the existence of hierarchy or seniority in the clinical clerkships. The hierarchy created inferiority and distance between juniors and seniors, and also between students and residents or preceptors/attending physicians. Respondents also perceived that the hierarchical culture led to different treatment and approaches when dealing with professional dilemmas among physicians, students, or other healthcare workers.

*‘Considering that the person has worked longer there than us*, *we must respect him/her*.*’* [FGD 4–CY]

*‘There are still many people who treated people differently based on the relationship*. *For example*, *special treatment was received because someone is related to a particular senior*, *which makes it more complicated [if the student reported the dilemma]*.*’* [FGD 2–S]

## Discussion

This study used focus group discussions to discuss and explore how medical students cope and react when facing professional dilemmas. Other studies have used narrative and reflective writings to explore similar situations [14,27]. Given the collectivist culture, students tend to be more comfortable discussing sensitive issues such as professional dilemmas in groups rather than individually, especially as the issues occurred in a learning situation in which students were afraid of the impact of personally speaking up towards their grades [18]. In addition, reflective ability and level of familiarity with narrative reflection among Indonesian health profession students were varied [28], hence having professional dilemma discussed in a group may somewhat facilitate individual reflection.

Despite the use of focus groups instead of narrative writing, this study showed similar results to previous studies, which demonstrated that students had negative emotions when facing dilemmas [7,27]. The impact on negative emotions and concerns on facing professional dilemmas might be due to the relationships between emotion and memory. It has been argued that emotional experiences are indelible and remembered accurately [29,30].

Emotions mentioned by students in facing professional dilemmas had caused them to avoid similar situations, regardless of the learning context. This finding is related to the self-determination theory showing that motivation is dynamic, and the learning environment may affect students’ motivation. The existence of a professional dilemma in clinical clerkship caused students to feel demotivated and avoid similar situations due to the failure in fulfilling their basic needs, especially the sense of relatedness [31]. As students tend to avoid such situations, teachers should pay close attention, especially to students who use avoidant coping strategies, since they are at risk of experiencing mental health problems and possibly being left out from teaching and learning process [32].

As negative emotions such as fear, frustration, and anger were mentioned by respondents of this study, it showed how professional dilemma may lead to moral distress when students felt powerless or unable to take the appropriate action [33]. If this occurred and was repeated over a long period, moral distress led to moral residue, which would seriously compromise individuals’ thoughts and views of self, thus damaging their careers as health professionals [34].

Furthermore, we found that students comply to the dilemmatic situation as a form of response was more common than resistance. As Indonesia has a cultural dimension of high power distances, in which more senior or superior people have significant roles in making decisions or determining approaches in the environment, compliance as a response to a dilemma from medical students may occur more frequently [18]. This was also supported by our findings that hierarchical relationships in the workplace affected how students responded towards dilemma. As students felt inferior and in a much lower position within the hierarchy, students tended to refuse showing disapproval, even if they faced things that were against their values and conscience [19,20].

Our findings also showed that resistance against the dilemma was the least described response to professional dilemmas from medical students. Rees et al. (2013) also found similar results, that only a few students reported mistreatment and showed resistance in the face of dilemmas [27]. Perceptions, experiences, and interactions within the clinical and educational institutions influence the medical students’ final decisions regarding whether or not to report the situation. This was in line with our findings that existence of reporting and follow-up mechanism as well as perceptions of dilemma among stakeholders has become factors on how students responded towards dilemma [35].

Shaw et al. (2018) identified several reasons underlying medical students’ resistance to dilemmas: to relieve the patients’ and/or students’ distress, to avoid the unqualified service, and to make the perpetrator aware that the action was wrong [36]. It was interesting to find that in our findings, students would dismiss their willingness to report, unless the behaviour was really unacceptable and there were victims. Perceptions about dilemma and professional behaviours in our finding was somehow stretched to what was commonly conducted in the workplace, hence affecting greatly on how students responded towards dilemma.

Besides the socialization process in the workplace, this study also highlighted how perceptions towards dilemma and (un)professional behaviours were also affected by ones’ cultural background and how ones were raised. Community in high power distance society tends to expect their children and students to be obedient, parents and teachers were more sovereign to their children or students than in lower power distance society [37]. Hence, ones tend to tolerate any behaviours shown by anyone more superior as forms of respect, therefore, affecting their perceptions towards dilemma and also how they respond towards them.

The collectivistic culture was also reflected in our finding as students decided not to resist against dilemmas because they were not only concerned about the consequences for themselves, but also for the group. They were also afraid that their actions, especially when resisting against dilemma, would eventually risk their present and future careers, individually and in groups. This phenomenon also reflected high uncertainty avoidance culture in which people prefer more towards security and stability in careers [21].

This study has further implications for medical education. It was known that students’ professional development should be nurtured through an adequate socialisation process in the workplace [38], where professional dilemmas might commonly take place. Professional dilemmas, therefore, can be regarded as venues to develop medical students’ professionalism, especially when they are discussed explicitly [39]. Our findings show that factors affecting how students responded towards dilemma emphasized on the needs of more authoritative roles in the institutions which could provide greater psychological support for students to promote their well-being [40]. It is important to provide formal sessions in which students feel psychologically safe and comfortable to discuss significant dilemmatic experiences or unfavourable role modelling [41,42]. Engagement in reflective dialogues within peer groups and mentors that enabled students to share their concerns or mistakes, learn from the experiences, and support their resilience along with their professional identity formation was also highlighted [43–48]. Role playing activities are also recommended to help students express and enact the ideal situation or what would they have wanted in order to support them to commit to professionalism [49].

In addition, development of curriculum, institutional policy, and academic health systems should be attempted to balance between providing supports for students’ coping against dilemma (through creating conducive environment, building clear reporting and follow up mechanism, etc.) and promoting students’ professional development (through engaging in reflective discussions and group mentorships, learning from negative role models) during clinical clerkships [50,51]. Furthermore, as teaching professionalism still have its own challenges, faculty development program on this particular area especially in the clinical clerkship program should regularly be held to promote professionalism not only for faculty individual level but also for a more positive cultural change in the institutional level [2].

There are some limitations in this study that need to be taken into account. First, the study was conducted in only one medical school in Indonesia; hence, the medical students’ responses and influencing factors reported in this study could be institution-specific. We were also aware of the possibility that cultures of particular clinical disciplines, aside from individual values, may affect how dilemmatic events and responses towards dilemma were perceived by students. However, it was beyond the scope of this study as our aim was to explore more about the responses towards dilemmas. Second, we employed only FG method to collect data, thus further studies on responses towards professional dilemma could focus on triangulation using other methods, such as questionnaire and reflective journaling. Third, due to consideration of sensitive issues reported in this study, we could not include further details on clinical rotations, teaching hospitals, or health professions in this paper. However, we still aimed to describe the students’ expressions as clearly as possible, which enabled discussions on their responses to professional dilemmas rather than ‘pointing fingers’ at whom to blame.

We would like to emphasise the responses and explore whether such responses were influenced by the sociocultural values in the society, as depicted in the objective of this study. Of course, we realise that supporting medical students in dealing with professional dilemmas requires a comprehensive approach that considers all factors: the people (medical students, clinical teachers, residents, other health professions) and their interactions, as well as the overall healthcare and clinical education systems. We should also acknowledge that medical students carried the sociocultural values within themselves, hence they should be empowered to learn from the dilemma while institutions prepare the systems to create more supportive learning environment [7,41,42]. Further studies exploring how students learn from dilemma and factors associated with the responses towards dilemma in different sociocultural context would be useful to enrich our understanding on this particular issue.

## Conclusions

This study has identified that sociocultural values, to some extent, influenced the way medical students responded to professional dilemmas in the workplace. Feelings of concerns/distress and compliance to the dilemma dominated the responses, which suggest that high power distance, high uncertainty avoidance, and collectivist culture have somewhat influenced student behaviour. Therefore, it is paramount to develop a conducive and culturally sensitive educational environment and nurture in students the ability to learn from the professional dilemma experienced in the workplace, as informed by this study findings, for students to develop their professional identity.

## Supporting information

S1 File Impact, response, and factors related to professional dilemmas in medical students. (DOCX)
