# Supplementary material for: “To obey or not to obey” - Medical students’ response towards professional dilemmas in a hierarchical and collectivist culture
Source: PLoS One. 2021 Dec 23;16(12):e0261828. doi: 10.1371/journal.pone.0261828 (PMC8699955; doi:10.1371/journal.pone.0261828)
Supplement: S1 File — (DOCX) [file pone.0261828.s001.docx]

**Table 1. Impact, response, and factors related to professional dilemmas in medical students**

| **No** | **Identified themes and subthemes** | **Numbers of quotes** |
| --- | --- | --- |
| 1 | Impact of dilemmas on students’ emotions and concerns | 72 |
| 2 | Responses towards professional dilemmas |  |
|  | 1. 1. Compliance | 54 |
|  | 2. Resistance | 44 |
| 3 | Factors affecting responses to dilemmas: |  |
|  | 1. Reaction of authority figures towards dilemmas | 29 |
|  | 2. Existence of clear reporting and follow-up mechanism | 27 |
|  | 3. Future implications of students’ responses | 21 |
|  | 4. Perceptions of dilemma and professionalism | 33 |
|  | 5. Hierarchical relationships in the workplace | 22 |
